# Supplementary material for: Intensifying tropical cyclones in the Arabian Sea replenish depleting aquifers
Source: Commun Earth Environ. 2025 Jul 8;6(1):536. doi: 10.1038/s43247-025-02493-w (PMC12234357; doi:10.1038/s43247-025-02493-w)
Supplement: Supplementary file 1 — Supplementary Information [file 43247_2025_2493_MOESM1_ESM.pdf]

Supplementary Information for

Intensifying tropical cyclones in the Arabian Sea replenish depleting aquifers

Hassan Saleh[1], Mohamed Sultan[1*], Eugene Yan[2], Himanshu Save[3], Hesham Elhaddad[1,4], Hadi Karimi[1], Karem Abdelmohsen[4,5], Mustafa K. Emil[1], Sara Al Qamshouai[6]

1 Department of Geological and Environmental Sciences, Western Michigan University, Kalamazoo, MI 49008, USA
2 Environmental Science Division, Argonne National Laboratory, Argonne, IL 60439, USA
3 Center for Space Research, University of Texas at Austin, Austin, TX 78759, USA
4 Geodynamics Department, National Research Institute of Astronomy and Geophysics (NRIAG), Helwan, Cairo, 11421, Egypt
5 School of Sustainability, Arizona State University, Tempe, AZ 85281, USA
6 School of Sustainable Engineering and the Built Environment, Arizona State University, Tempe, AZ 85281, USA

* Corresponding author

Table of Contents

Supplementary Notes 2
Supplementary Figures 5
Supplementary Tables 11
Supplementary References 14

Supplementary Notes

Supplementary Note 1: Study region

The southern sections of the AP are bound by mountain chains, the Hajar Mountains from the east, the Dhofar Mountains from the south, and the Sarawat Mountains from the west (Supplementary Figure 1). The population is concentrated in a narrow coastal zone that sits between the Arabian Sea and the mountain chains. The Najd subbasin covers an area of 200,000 km^2 (Supplementary Figure 1) in Oman, Yemen, and Saudi Arabia and extends from the southern Dhofar Mountains (up to 1800 m.a.s.l) in the south to the dune fields (up to 58 m.a.s.l) in the North.

The climate in the Najd subbasin ranges from semi-arid on the coast to hyper-arid in the north, where temperatures of up to 50°C are recorded in the Rub Al Khali desert[1]. The average annual precipitation is 30 mm yr^{-1} and ranges from <30 mm yr^{-1} in the north to 70 mm yr^{-1} south of Najd[2]. Precipitation occurs in three seasons: the monsoon, winter, and cyclone. Monsoons start in mid-June and end in mid-September. They originate in the Arabian Sea and drive moist air to the coastline. Monsoon precipitation is up to 300 mm yr^{-1} and is restricted to the mountain's coastal side. However, it does not affect the Najd subbasin and its underlying aquifers on the northern side of the mountains[3]. In the winter season (December-April), north-westerly frontal systems from the Mediterranean or the Red Sea bring light precipitation[4] that often evaporates before or even after reaching the water table in shallow aquifers[5]. The cyclone season occurs during the pre-monsoon (May-June) and post-monsoon (October-November) and brings extreme precipitation that lasts for days over the Najd subbasin[4]. At the same time, potential evaporation is high, reaching 2200 mm yr^{-1} in the interior region[3].

The Najd terrain is partly covered by a stony and sandy plain and dissected by wadi channels filled with alluvial deposits. The wadis originate from the Dhofar mountains, align with the regional slope, and act as ephemeral streams that channel surface runoff from extreme TCP for long distances (> 200 km) towards the dune fields in the lowlands (Supplementary Figure 2a).

Alluvium and dune deposits overlie Tertiary carbonates (Fars and Hadhramaut Groups). The Hadhramaut, but not the Fars Group or the alluvium, is targeted for groundwater production because of its larger average thickness (Hadhramaut: 542 m; Fars: 20 m; alluvium: 4 m) and its widespread distribution within the Najd[1]. The Hadhramaut Group comprises carbonates of the Lower Eocene to Paleocene Umm Er Radhuma (UER) Fm., the Lower Eocene Rus Fm., and the Middle to Lower Eocene Dammam Fm. (Supplementary Figure 2a)[3]. The thick (up to 900 m) Tertiary carbonates are separated from the underlying successions by the impermeable Cretaceous Shammar shale Fm. of unknown thickness[4].

The groundwater system comprises four aquifers (A, B, C, D; Supplementary Figure 2b) separated by thin (<3 m) shale layers[3]. Aquifer A (Dammam and Rus Fms.) is an unconfined to semi-confined aquifer in the central and northern sections of the Najd subbasin, whereas aquifers B, C, and D (UER Fm.) are confined aquifers[2]. Aquifer B underlies the central and western Najd, Aquifer C does not extend to the Dhofar Mountains, and only Aquifer D crops out in the mountains, where modern recharge occurs. Confined aquifers are primarily recharged at their southern mountain outcrops, where intense rainfall intersects highly karstified and fractured carbonate formations. Subsequently, groundwater discharges at Umm as Samim sabkha and dispersed springs northeast of the Najd (Supplementary Figure 2a)[4,6,7]. Groundwater levels in aquifers C and D are higher than those in aquifers A and B, indicating no leakage from the upper to the lower aquifers except where cones of depression occur in the lower aquifers[2].

Supplementary Note 2: Estimating Recharge and Pressure Front Transient Time

Isotopic tracers are not ideal for estimating recharge over short periods during which TCs are active[8]; the lack of data (streamflow, lysimeter, seepage meter, and water table fluctuations) hinders applications of physical methods and rainfall-runoff lumped or semi-distributed models[7, 8]. While estimating storage change using specific yield (for unconfined aquifers) and storativity (for confined aquifers) with measured water level changes provides a potentially valuable independent validation, this approach was not applied here due to substantial uncertainty in the hydraulic parameters[2]. Reported values for specific yield in the study area vary widely from 0.4% to 10%[1], and transmissivity values span several orders of magnitude, from tens to over 10,000 m^2 $day^{(-1)}$[9]. Moreover, aquifer designations at monitoring wells are often poorly documented, leading to uncertainties in hydraulic properties and groundwater level variations within each aquifer. Given these challenges, we recommend future field studies to obtain site-specific aquifer parameters, enabling robust validation of storage estimates.

We estimated the transient time for a pressure front to propagate in the confined aquifers from the recharge areas to the central Najd, where heavy pumping occurs. The pressure front equation[10] (Eq. S1) was applied using the average transmissivity T and storativity S of aquifers B and C, with a travel distance r of 50 km[1].

$$t_{PF}(r) = \frac{r^2}{4\pi} \times \frac{S}{T} \qquad \text{Eq. S1}$$

Supplementary Note 3: Soil Evaporation Capacitance (SEC) Model

There are two stages for soil evaporation. In stage (1), water is supplied to the evaporation surface by capillary flow, which is characterized by a high and relatively constant evaporation rate (Eq. S2)[11]. At the same time, water is also removed from the soil by leakage beyond the evaporation active depth (Eq. S3). During stage (2), evaporation occurs when vapor diffusion advances evaporation by disrupting capillary pathways (Eq. S4). The infiltration map from each TC simulation was used as the actual water content θ, which determines the evaporation stage. We calculated the characteristic length and multiplied the results by the saturated water content θ_{sat} and cell area to estimate maximum storage. In case the infiltration was more than the soil moisture capacity, it was assigned maximum storage and then updated daily by removing evaporation and leakage. The hydraulic properties of those soil types and how they were inserted in the equations below were described in detail by Lehmann et al. (2018)[12].

Soil evaporation in stage (1) was estimated using the following parameters that include potential evaporation rate ET_0, the hydraulic conductivity of the unsaturated capillary flow region which can be estimated as $4K(\theta_{crit})$ with hydraulic conductivity K of the soil at critical water content θ_{crit} and unsaturated hydraulic conductivity K(θ).

$$Es = \frac{ET_0 \cdot K(\theta)\left[1+\frac{ET_0}{4K(\theta_{crit})}\right]}{ET_0+K(\theta)\left[1+\frac{ET_0}{4K(\theta_{crit})}\right]} \qquad \text{Eq. S2}$$

Leakage is considered to occur only when mean water content θ exceeds a critical value θ_{crit}. The volume of drained soil water F(t) is estimated below as t is the elapsed time since the onset of capacitor leakage (marked by the end of the rainfall), and the hydraulic conductivity of the surface evaporation capacitor K(θ) and L_C the characteristic length.

$$F = L_C(\theta - \theta_{crit})\left[1 - exp\left(-\frac{t \cdot K(\theta)}{L_C(\theta - \theta_{crit})}\right)\right] \qquad \text{Eq. S3}$$

In stage (2), accumulative soil evaporation CE(t) is estimated as a function of the time (t) with change $\Delta\theta$ in water content above and below the vaporization front, evaporation rate E2, and a small value of vaporization plane "jump" or depth ξ at the onset of stage-II at time t_{II}. The evaporation rates E_2 at the onset of stage (2) are considered the minimum between 2.0 mm/day and $ET_0/2$, assuming a drying front at a depth of ξ =10 mm[13].

$$CE(t) = \frac{\sqrt{\xi}}{\sqrt{\xi + \frac{2E_2(t-t_{II})}{\Delta\theta}}}(2E_2(t - t_{II}) + \Delta\theta \cdot \xi) - \Delta\theta \cdot \xi \qquad \text{Eq. S4}$$

Supplementary Figures

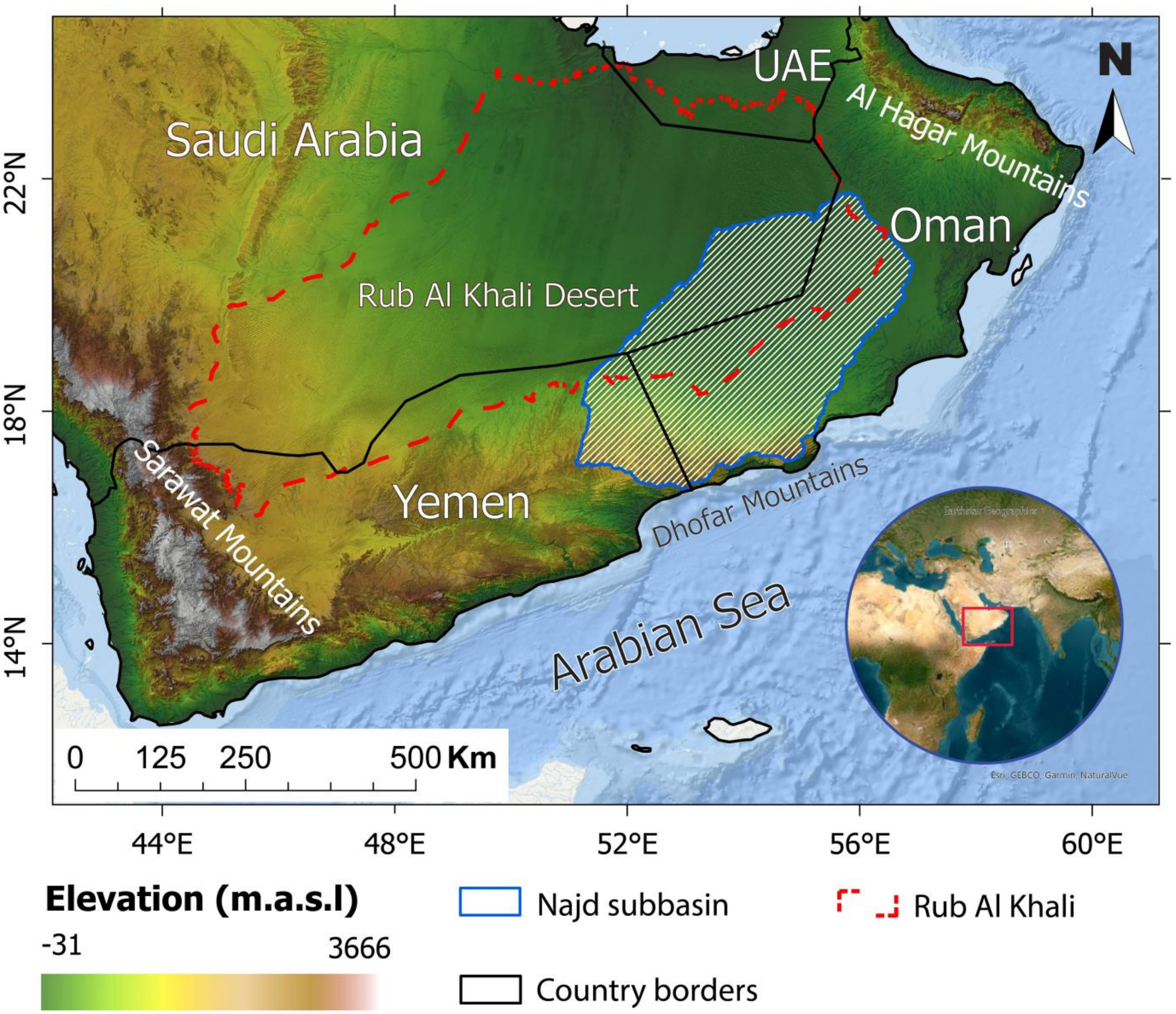

Supplementary Figure 1. Location map. Color-coded digital elevation map (DEM) for southern AP showing the distribution of the Red Sea Hills (west), the Hajar Mountains (east), the Dhofar Mountains (south), and the Sarwat Mountains (southwest). Also shown is the Najd subbasin extending from the Dhofar Mountains and comprising part of the Rub Al Khali desert.

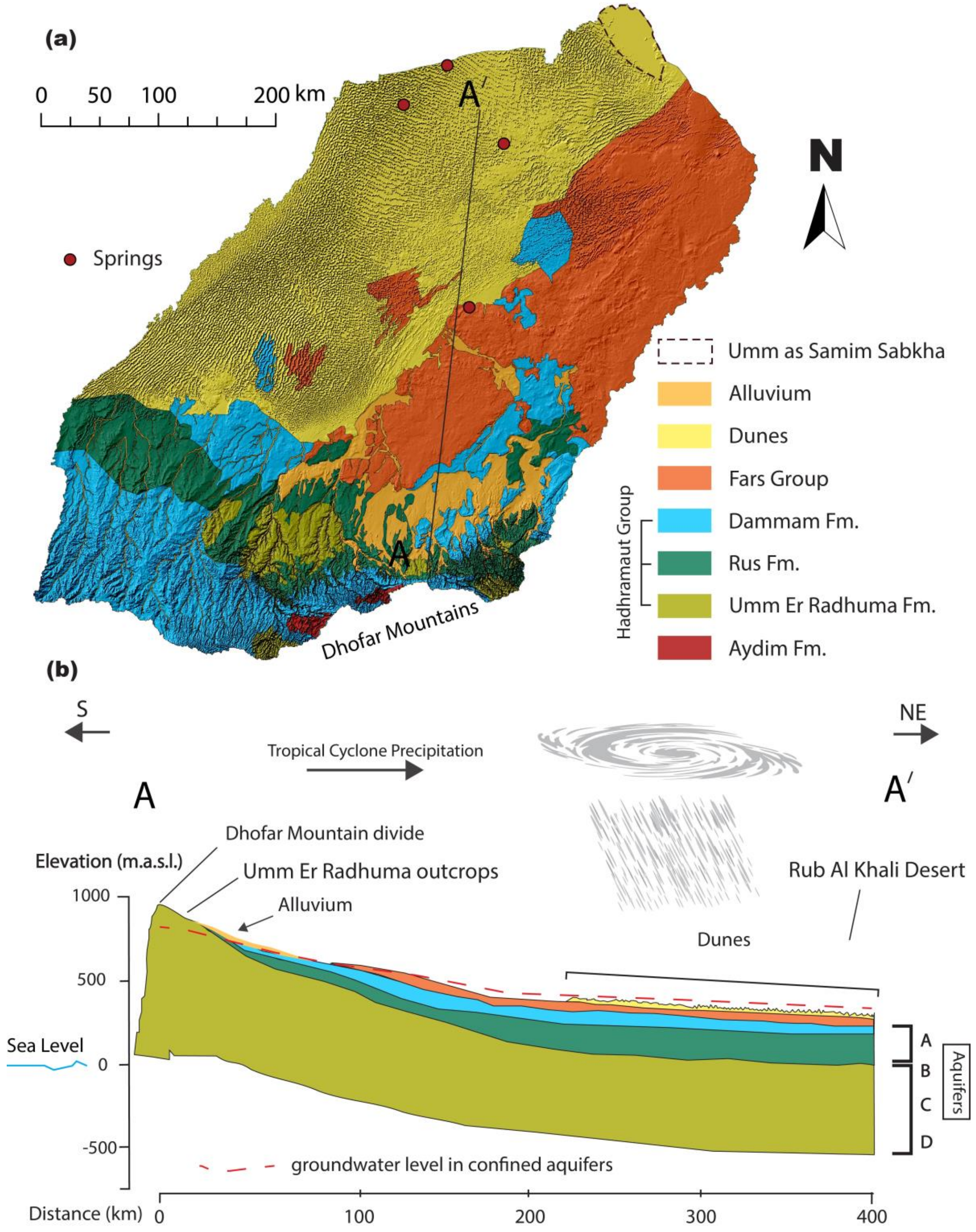

Supplementary Figure 2. Geologic and Hydrologic Setting. (a) Simplified geologic map of the Najd subbasin, after Al-Mashaikhi et al. (2012)[1] and van der Gun (1995)[14], overlays a hillshade (x15 vertical exaggeration) extracted from the DEM. The elevation decreases from 1825 m.a.s.l. in the southwest to 65 m.a.s.l. in the northeast. Groundwater recharge occurs at the outcrops of the UER Fm. and discharges at the Umm as Samim Sabkha. Carbonates cover most of the south and southeast parts of the subbasin. Wadis dissect the terrain, which originate from the mountains and extend northeast toward the dune field. (b) Schematic SW–NE trending cross-section along transect A–A’ in Supplementary Figure 2a modified from Al-Mashaikhi et al. (2012)[1] and Müller et al. (2016)[2].

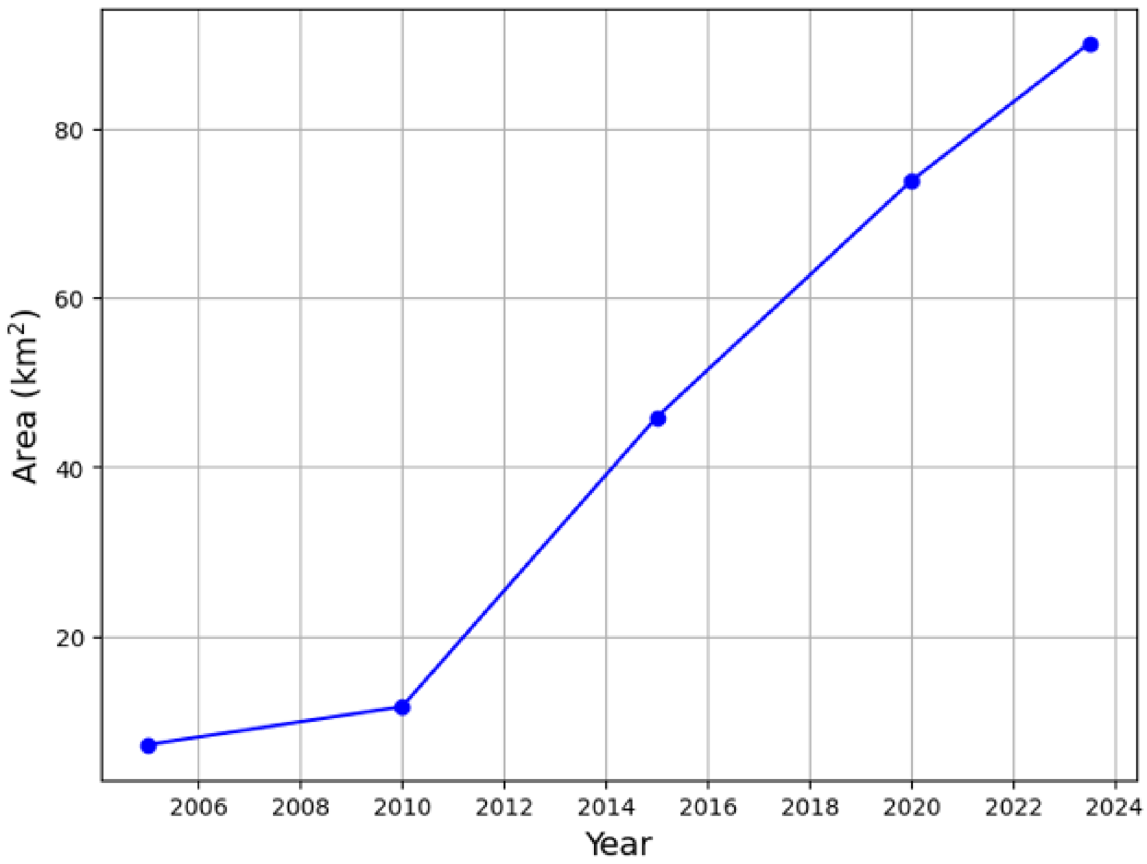

Supplementary Figure 3. Area of cultivated land. Data is extracted from the Normalized Difference Vegetation Index (NDVI) using Landsat-8 multispectral data L2 C2 on Google Earth Engine, sampled every 5 years over the Najd subbasin.

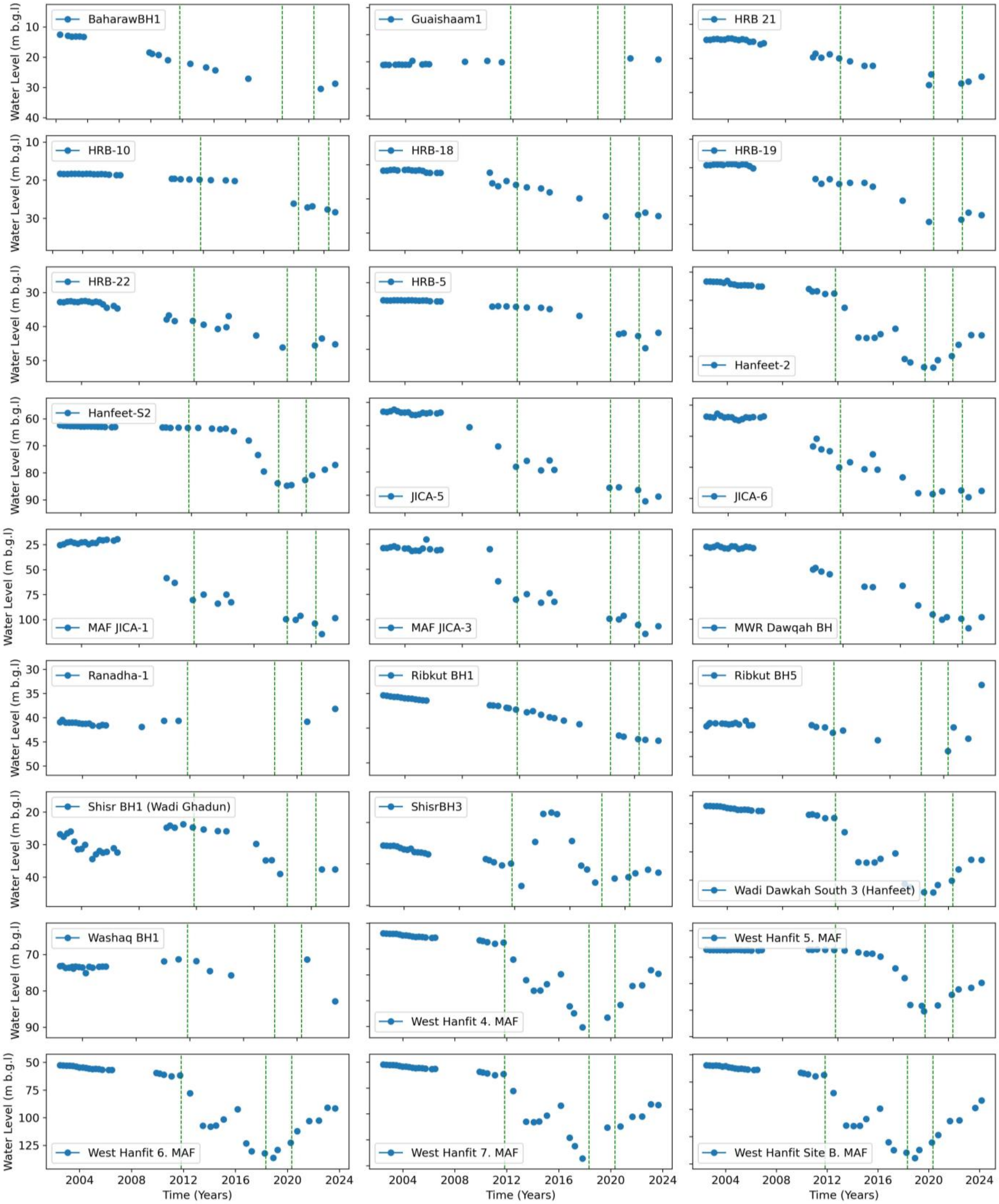

Supplementary Figure 4. Monitoring wells in the Najd Subbasin. Groundwater levels (meters below ground level) from available monitoring wells in the confined aquifers (C & D).

144

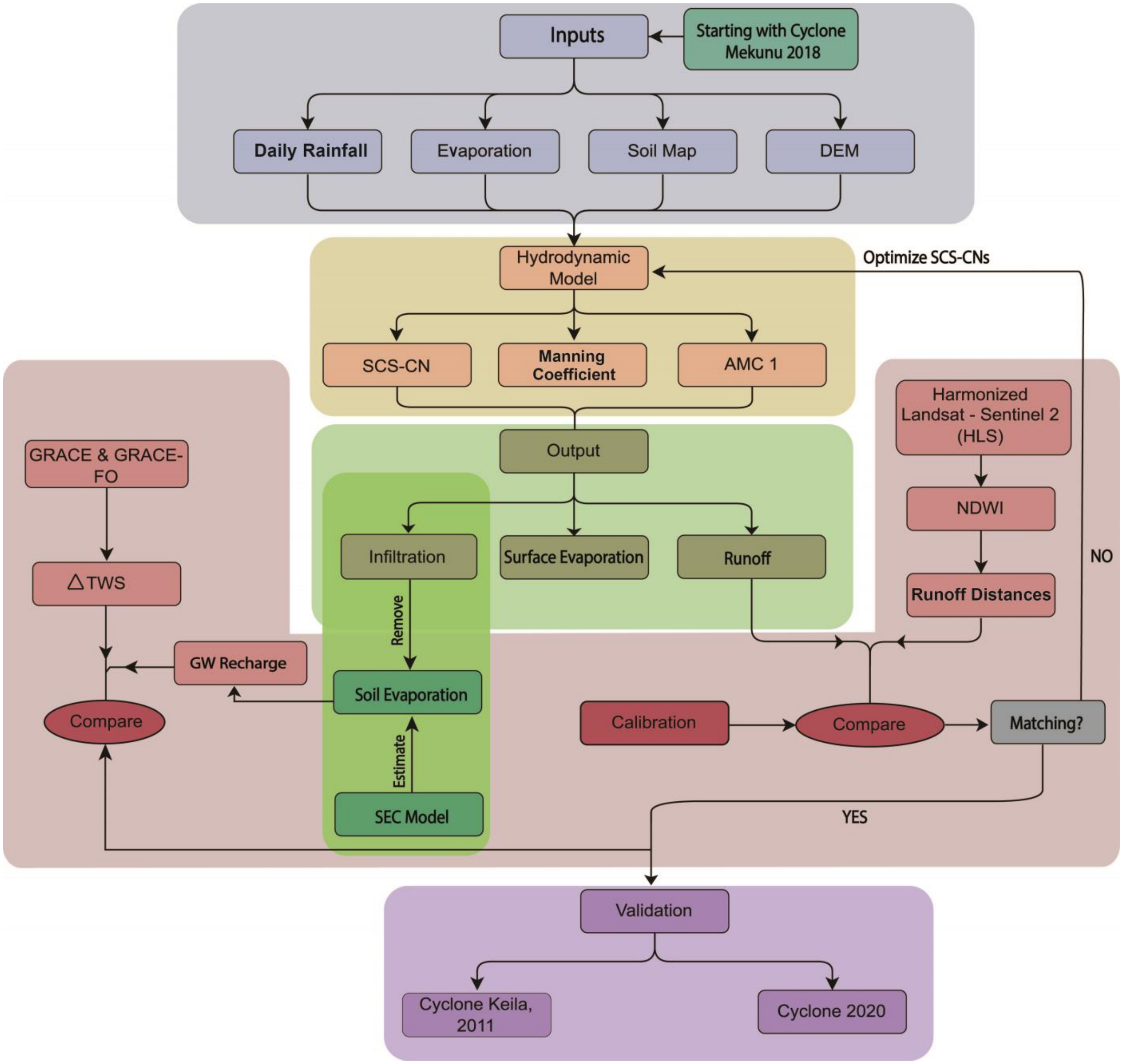

Supplementary Figure 5. Flowchart of the hydrodynamic model setup and comparison with $GRACE_{TWS}$. The model was set up for cyclone Mekunu with inputs that include static layers (DEM and soil types) and fluxes (rainfall and evaporation). The SCS-CN method was used for estimating rainfall partitioning with a Manning's coefficient of 0.035 and AMC 1 for the dry conditions. The runoff output was used for model calibration by adjusting the CNs iteratively. The model was then validated by simulating Cyclones Keila and 2020. Soil evaporation estimated by the SEC model was subtracted from the modeled infiltration to estimate recharge. The modeled recharge was then compared with recharge estimates from $GRACE_{TWS}$.

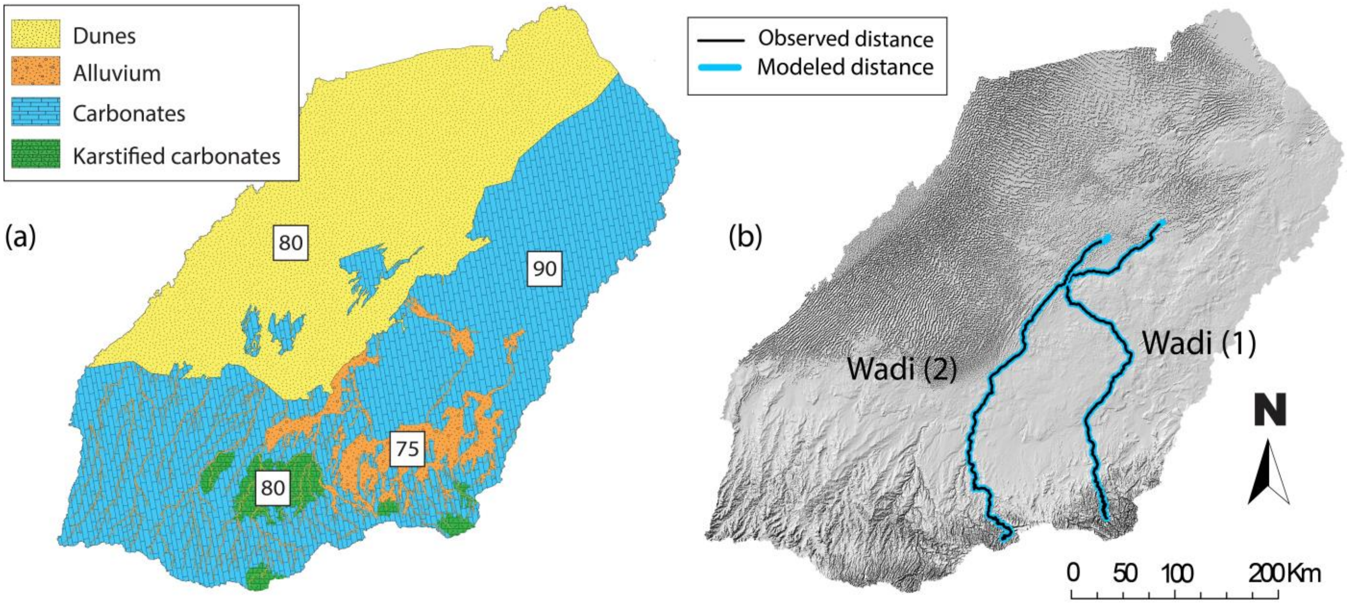

Supplementary Figure 6. Model inputs and calibration theme. (a) Soil types and their corresponding CNs are assigned using information extracted from geologic maps and soil cover[1,14]. (b) Observed and modeled runoff traveling distances downstream from the mountain divide along two main wadis following the landfall of cyclone Mekunu (2018).

Supplementary Tables

Supplementary Table 1: List of TCs that made landfall in the AP (1990 – 2020). Cyclone categories are based on the WMO regional classification. The landfall dates are extracted from the first record of TC's intersection with AP.

Name	Year	Landfall Date	Dissipation Date	Category	MSW (km/h)
NOT_NAMED	1992	1992-10-03 00:00	1992-10-04 09:00	Cyclonic Storm	83
NOT_NAMED	1994	1994-06-09 12:00	1994-06-09 15:00	Severe Cyclonic Storm	102
NOT_NAMED	1998	1998-12-17 06:00	1998-12-17 15:00	Severe Cyclonic Storm	102
NOT_NAMED	2002	2002-05-10 12:00	2002-05-10 15:00	Cyclonic Storm	65
GONU	2007	2007-06-05 21:00	2007-06-07 21:00	Super Cyclonic Storm	235
NOT_NAMED	2008	2008-10-23 15:00	2008-10-23 15:00	Depression	56
PHET	2010	2010-06-03 21:00	2010-06-07 03:00	Very Severe Cyclonic Storm	157
KEILA	2011	2011-11-03 03:00	2011-11-05 03:00	Cyclonic Storm	65
CHAPALA	2015-a	2015-11-03 00:00	2015-11-03 21:00	Extremely Severe Cyclonic Storm	213
MEGH	2015-b	2015-11-10 06:00	2015-11-10 09:00	Extremely Severe Cyclonic Storm	176
MEKUNU	2018-a	2018-05-25 18:00	2018-05-27 03:00	Extremely Severe Cyclonic Storm	176
LUBAN	2018-b	2018-10-14 03:00	2018-10-14 21:00	Very Severe Cyclonic Storm	139
HIKAA	2019	2019-09-24 12:00	2019-09-26 03:00	Very Severe Cyclonic Storm	139
NOT_NAMED	2020	2020-05-29 12:00	2020-05-31 15:00	Depression	46

Supplementary Table 2: Summary of $GRACE_{TWS}$ changes after the landfall of cyclones Keila, Mekunu, and 2020 from three mascon solutions (CSR, JPL, GSFC), their mean, and standard deviation in km^3.

Cyclone	Solution	One-Year Difference Detrended
Keila 2011	CSR	-0.1
	JPL	0.3
	GSFC	0.0
	Mean	0.1
	STD	0.2
Mekunu 2018	CSR	5.4
	JPL	1.4
	GSFC	3.3
	Mean	3.4
	STD	1.6
2020	CSR	0.9
	JPL	-0.2
	GSFC	-1.0
	Mean	-0.1
	STD	0.8

Supplementary Table 3: Selected satellite imagery scenes that captured the runoff dynamics following the landfall of cyclones Keila, Mekunu, and 2020 in the Najd.

Cyclone	Time	Satellite/Product
Keila 2011	2011-11-10 - 7:00 UTC	Landsat 7 ETM+ L2
Mekunu 2018	2018-05-29 - 7:00 UTC	Harmonized Landsat Sentinel
	2018-06-03 - 7:00 UTC	Harmonized Landsat Sentinel
	2018-06-08 - 7:00 UTC	Harmonized Landsat Sentinel
2020	2020-06-03 - 7:00 UTC	Harmonized Landsat Sentinel
	2020-06-07 - 7:00 UTC	Harmonized Landsat Sentinel
	2020-06-12 - 7:00 UTC	Harmonized Landsat Sentinel

Supplementary Table 4: Simulation time for three modeled TCs

Cyclone	Simulation start date	Simulation end date	Number of days
Keila 2011	October 26, 2011	November 13, 2011	18
Mekunu 2018	May 23, 2018	June 9, 2018	17
2020	May 26, 2020	June 12, 2020	17

Supplementary Table 5: Runoff travel distances in km from the hydrodynamic model and the available satellite imagery in two major wadis following the landfall of cyclone Keila (2011), cyclone Mekunu (2018), and cyclone 2020.

Cyclone	Date	Observed		Modeled	
		Wadi (1)	Wadi (2)	Wadi (1)	Wadi (2)
Keila 2011	10-11-2011	383.0	306.2	369.0	328.1
Mekunu 2018	29-05-2018	391.6	431.7	442.9	387.6
	03-06-2018	459.9	432.8	458.6	428.8
	08-06-2018	468.8	438.3	472.7	447.9
2020	03-06-2020	355.4	278.8	281.7	279.4
	07-06-2020	446.6	294.2	372.5	277.6
	12-06-2020	451.2	392.8	280.6	294.2

Supplementary Table 6: Relationship between Curve Numbers and corresponding soil types in Najd

Lithology	Curve Number	Soil Group	Soil Type
Alluvium	75	A	LOAMY SAND
Dunes	80	B	SANDY LOAM
Carbonates	90	C	LOAM
Karstified Carbonates	80	B	SANDY LOAM

Supplementary Table 7: Hydraulic properties estimated for three field sites using sand and clay fractions[12,13].

Site	Sand %	Clay %	Soil Type	θ_{res}	θ_{sat}	(1/m)	n (-)	K_{sat} (m/d)
USDk1	48	9	LOAM	0.0406	0.3966	1.11	1.4977	0.2884
USMo1	66	10	SANDY LOAM	0.0416	0.3860	3.15	1.4061	0.4092
USMo7	80	6	LOAMY SAND	0.0411	0.3847	4.03	1.6809	0.9430

Supplementary References

1. Al-Mashaikhi, K. *et al.* Evaluation of groundwater dynamics and quality in the Najd aquifers located in the Sultanate of Oman. *Environ. Earth Sci.* **66**, 1195–1211 (2012).
2. Müller, Th. *et al.* Use of multiple age tracers to estimate groundwater residence times and long-term recharge rates in arid southern Oman. *Appl. Geochem.* **74**, 67–83 (2016).
3. Al-Mashaikhi, K. Evaluation of groundwater recharge in Najd aquifers using hydraulics, hydrochemical and isotope evidences (Doctoral Thesis). (Friedrich-Schiller-Universität Jena, Germany, 2011).
4. Muller, T. Recharge and residence times in an arid area aquifer (Doctoral thesis). (Technische Universität Dresden, Dresden, 2012).
5. Schulz, S. *et al.* Groundwater evaporation from salt pans: Examples from the eastern Arabian Peninsula. *J. Hydrol.* **531**, 792–801 (2015).
6. Sultan, M. *et al.* Geochemical, isotopic, and remote sensing constraints on the origin and evolution of the Rub Al Khali aquifer system, Arabian Peninsula. *J. Hydrol.* **356**, 70–83 (2008).
7. Fookes, P. G. & Lee, E. M. Desert environments of inland Oman. *GeologyToday* **25**, 226–231 (2009).
8. Scanlon, B. R., Healy, R. W., Scanlon, B. R., Healy, R. W. & Cook, P. G. Choosing appropriate techniques for quantifying groundwater recharge. *Hydrogeol. J.* **10**, 18–39 (2002).
9. Clark, I. D. *et al. Modern and Fossil Groundwater in an Arid Environment: A Look at the Hydrogeology of Southern Oman*. 167–187 (1987).
10. Chesnaux, R. Avoiding confusion between pressure front pulse displacement and groundwater displacement: I llustration with the pumping test in a confined aquifer. *Hydrol. Process.* **32**, 3689–3694 (2018).
11. Lehmann, P., Assouline, S. & Or, D. Characteristic lengths affecting evaporative drying of porous media. *Phys. Rev. E - Stat. Nonlinear Soft Matter Phys.* **77**, (2008).
12. Lehmann, P., Merlin, O., Gentine, P. & Or, D. Soil Texture Effects on Surface Resistance to Bare-Soil Evaporation. *Geophys. Res. Lett.* **45**, 10,398-10,405 (2018).
13. Or, D. & Lehmann, P. Surface Evaporative Capacitance: How Soil Type and Rainfall Characteristics Affect Global-Scale Surface Evaporation. *Water Resour. Res.* **55**, 519–539 (2019).
14. Jac van der Gun. Geological Map of Yemen (Fig 2.3 in 'The Water Resources of Yemen'), Report Number: 209 WRAY-35. (1995).
